# Tooth brushing practice in Ethiopia: a systematic review and meta-analysis

**DOI:** 10.1038/s41598-023-33541-0

**Published:** 2023-04-19

**Authors:** Addisu Tadesse Sahile, Mitiku Tesfaye Wondimu, Endeshaw Mulate Fikrie

**Affiliations:** 1 Department of Public Health, Unity University, Addis Ababa, Ethiopia; 2 Department of Dentistry, Menelik II Referral Hospital, Addis Ababa, Ethiopia; 3 Department of HIV/AIDS Prevention, Addis Ababa, Health Bureau, Addis Ababa, Ethiopia

**Keywords:** Diseases, Health care, Medical research

## Abstract

Oral hygiene refers to taking care and maintaining the cleanness of gum and teeth; a good oral hygiene practice promotes better oral health in general. Oral hygiene is the top public health concern of the population. Tooth brushing is a technique to keep oral hygiene from related complications. Therefore, this study provides the pooled prevalence of tooth brushing practice in Ethiopia. Databases searched for articles systematically across PubMed, Google Scholar, Hinari, EMBASE, and African Journals Online. Two reviewers independently conducted the selection, screening, reviewing, and data extraction using a Microsoft Excel spreadsheet and used the Joanna Briggs Institute prevalence critical appraisal tools to assess the quality of evidence. All studies conducted in Ethiopia from 2010 to 2020, reporting tooth-brushing practices extracted for and imported into the Comprehensive meta-analysis version 3.0 for further analysis. Beggs and Eggers’s tests evaluated for publication bias with Higgins’s method evaluated for heterogeneity. A random-effects meta-analysis model with a 95% confidence interval was computed to estimate the pooled effect size (prevalence). Furthermore, the authors employed subgroup analysis based on the study area and sample size. After reviewing 36, 10 articles fulfilled the inclusion criteria, and were included in the meta-analysis. The pooled prevalence of tooth brushing practice was 12.2% (95% CI 7.6–19.2%). The review reported a lower level of tooth-brushing practice in Ethiopia. We recommended that special attention should be given to the oral hygiene of the Ethiopian people.

## Introduction

According to the global oral health status report for 2022, oral disease affected about 3.5 billion people worldwide, where three out of four affected people live in middle-income countries^1^. Moreover, dental caries of permanent teeth affected two million people worldwide^2^.

Dental caries is a progressive and irreversible degradation of the enamel/dentin following acid production as a result of bacterial metabolism^3^ and causes teeth’ mineralization and destruction of the teeth’ hard tissues^3,4^. It is one of the public health concerns of the world^5^, and nearly every adult in the world has dental caries^6^. In children, it is a more prevalent condition affecting 60–90%^7–9^.

With the growth of urbanization and changes in living conditions worldwide, the prevalence of main oral diseases continued to increase^10^. For this to exist, inadequate-water supply and oral hygiene products, poor access to oral health care by the communities and high sugar-containing foods availability, and affordability were among the factors that played a great role^11^.

Beyond affecting the quality of life^12^, poor oral health accelerates the risks of morbidity and mortality^13^. Dental caries is one of the determinant factors for poor oral health in a population, whose existence is either facilitated or determined by factors such as educational level, occupation, income^14–16^, PH, social classes, oral hygiene, viscosity, and buffer capacity of the saliva, carbohydrate diet, and parental incidence of caries^17^.

Oral hygiene refers to taking care and maintaining the cleanness of gum and teeth^18–20^ whereas the practice of it promotes better oral health^21–23^. The American Dental Association stated oral health as “a functional, structural, aesthetic, physiologic, and psychosocial state of well-being that is essential to an individual’s general health and quality of life”^24^.

Oral hygiene is the top public health concern of the world population^25^ same talk; the prevalence of oral diseases in Ethiopia was observed at up to 90% of the population^26^. Corpus of evidence reported the positive effect of tooth brushing at preventing and reducing the oral health conditions^27–32^.

To the best of the researcher’s knowledge, the level of tooth brushing practice in Ethiopia was not a well investigated and understood area; and hence this review examined the state of evidence on the level of tooth-brushing practice in Ethiopia. The review question is “What is the level of tooth brushing practice in Ethiopia?”.

## Methods

### Reporting

The preferred Reporting Items for Systematic Reviews and Meta-Analyses (PRISMA) guideline^33^ was used to report this meta-analysis (Additional File 1 research checklist).

### Searching strategies

The reviewers followed the PRISMA systematic review protocol as a reporting guideline for the PRISMA checklist, eligible studies for the study were selected in terms of titles, abstracts, and then full articles based on inclusion criteria. PubMed, EMBASE, Hinari, African Journals Online, and Google scholar systematically searched for based on controlled and free-text languages. In terms of free-text searches, the keywords included the followings: (Tooth Brushing OR Dental Problems OR Dental Caries) AND Ethiopia. The controlled searches included the following Medical Subject Heading (MeSH) terms: “tooth brushing”, and “Ethiopia” as recommended for each database. The search terms were used individually and in combination using “AND” and “OR” Boolean operators. Moreover, the search was guided by PICO, population- a population that practiced tooth brushing.

### Inclusion and exclusion criteria

The reviewers included the following type of studies (1) study population comprised any age group, (2) study outcome is tooth brushing, (3) study design is cross-sectional, and articles published in English language. Meanwhile, the reviewers excluded the following, (1) qualitative studies, (2) report language is non-English, and (3) year of publication is older than 2010.

### Outcome of interest

#### PICO

The population of the study was any age group that brushed tooth. The main outcome was the tooth brushing practice reported in the reviewed articles both as percentage and frequency, calculated by dividing the number of individuals who brushed their tooth twice or more times a day by the total sample size then multiplied by 100.

### Screening and data extraction

Two reviewers (SAT and WMT) screened titles and abstracts against the inclusion criteria and then did an independent assessment for full text articles based on the predetermined inclusion and exclusion criteria. Arguments discussed and reached on complete consensus. The three authors (SAT, WMT, and FEM) independently did the data extraction from a random sample of 20% to check consistency and found no variation.

### Study quality assessment

While designing a data abstraction form on Microsoft Excel, the reviewers emphasized for clarity of the data, objective, study design, population, year of publication, sample size, and proportion of tooth brushing (Table 1). An assessment for the methodological qualities was based on the Joanna Briggs Institute prevalence critical appraisal tool for the critical appraisal of the studies^34^.

**Table 1:** Characteristics of included studies, their sampling methods, and outcome.

Author, year	Study place	Sampling methods, and response rate	Study design	Sample size and age in years	Prevalence of tooth brushing
Shedev et al. (2020)^35^	Mekelle	Stratified sampling Response rate: NA	Cross- sectional	N:384 Age:15–20	5.5%
Meyrema and Kedir (2018)^36^	Adama	Systematic random sampling Response rate 100%	Cross-sectional	n = 422 Age: ≥ 20	46.0%
Shukure and Shuke (2017)^37^	Fitche	Simple random sampling Response rate: NA	Cross-sectional	N:264 Age: ≥ 16	24.3%
Darout (2014)^38^	Jimma	Convenience sampling Response rate: NA	Cross-sectional	N:266 Age: ≥ 18	30%
Gualie and Tayachew (2018)^39^	Debre Tabor	Systematic random sampling Response rate 100%	Cross-sectional	N:422 Age :NA	3.3%
Teshome et al. (2016)^40^	Finote Selam	Simple random sampling Response rate 100%	Cross-sectional	N:291 Age:12–20	31%
Kebede et al. (2012)^41^	Jimma	Simple random sampling Response rate: NA	Cross-sectional	N:240 Age:15–68	3.75%
Teshome et al. (2020)^42^	Gonder	Systematic random sampling Response rate :NA	Cross-sectional	N:368 Age: Any	6.5%
Shenkute and Asfaw (2019)^43^	Jimma	Convenience sampling Response rate : NA	Cross-sectional	N:115 Age :NA	6%
Dechasa et al. (2017)^44^	Addis Ababa	Sampling methods: NA Response rate 100%	Cross-sectional	N:384 Age: ≥ 20	19.5%
Abate et al. (2020)^45^	Addis Ababa	Simple random sampling method Response rate: NA	Cross-sectional	N:320 Age :NA	22.3%

### Data synthesis and statistical analysis

The extracted data using Microsoft Excel spreadsheet imported to Comprehensive meta-analysis version 3.0 software for further analysis. The pooled effect size with a 95% confidence interval of tooth brushing practice was determined using a weighted inverse variance random-effects model. The I^2^ statistic used for assessing the heterogeneity across the studies, where 25, 50 and 75% represent low, moderate, and high heterogeneities consecutively^46^. A funnel plot and Beggs and Eggers tests evaluated the risks of publication bias^47^.

## Results

### Selection of the studies

**Figure 1:**
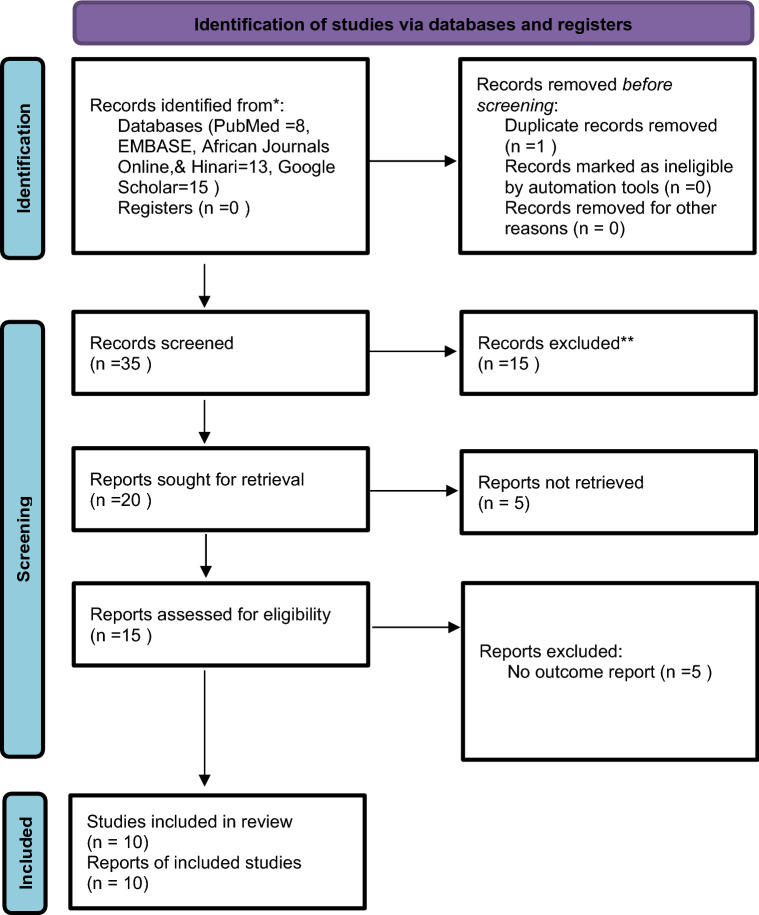
PRISMA flow diagram for showing screening and selection process of duties.

A comprehensive literature search for the databases yielded 36 published articles, of which eight articles retrieved from PubMed, 13 articles from EMBASE, African journals online, and Hinari, and 15 articles from Google Scholar. Twenty-one articles excluded once assessed for duplication. Fifteen articles screened by abstracts, of which five articles excluded for no outcome report. Ten full-text articles that fulfilled the eligibility criteria with a total sample size of 3156 included in the final analysis for the systematic review and meta-analysis (Fig. 1).

### Characteristics of the included studies

Pertinent information about authors, publication year, population, study area, sample size, age groups, outcome, and main findings from the selected articles were extracted and presented in Table 1. All articles were cross-sectional and in Ethiopia from 2010 to 2020 and published in indexed journals. The studies were conducted in Jimma^38,41,43^, Addis Ababa^44,48^, Adama^36^, Fitche^49^, Mekelle^35^, Gondar^42^, Fenote Selam^40^, and Debre Tabor^39^. The sample size for the selected studies ranges from 115 to 422 (Table 1).

### Tooth brushing practice

**Figure 2:**
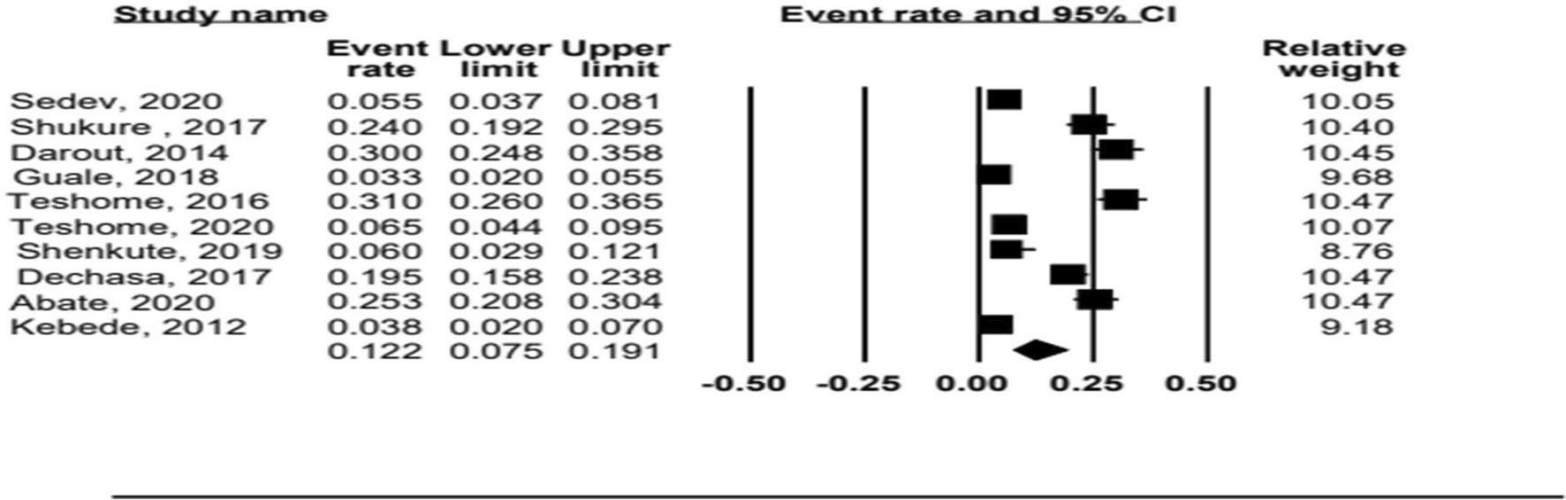
Forest plot of pooled prevalence/level/of tooth brushing practice.

In this systematic review and meta-analysis, the pooled estimate of tooth brushing practice was described by forest plot. The pooled prevalence of tooth brushing practice in Ethiopia from the random effects method observed was 12.2% (95% CI 7.6–19.2%) (Fig. 2).

### Assessment of publication bias

**Figure 3:**
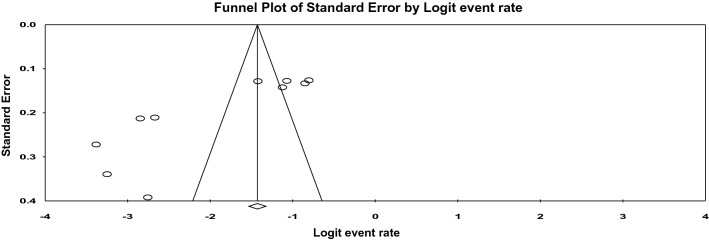
Funnel plot for the selected studies.

The funnel plot found to be asymmetry and Begg’s and Egger’s tests showed presence of a significant publication bias at a P-value of 0.05 (Fig. 3).

### Investigation of heterogeneity

For identifying the possible causes of variation across the studies, meta-regression analysis was done using sample size and study area. The result showed that there is no significant heterogeneity across the studies (P > 0.05) (Table 2).

**Table 2:** Sources of heterogeneity across the studies.

Source of heterogeneity	Coefficient	Standard error	I^2^ (%)	P-value
Sample size	− 0.0018	0.0061	99.0	0.7688
Study area	− 0.9998	− 0.8781	98.4	0.557

## Discussion

This systematic review and meta-analysis aimed at providing comprehensive evidence on the level of tooth brushing practice in Ethiopia from 2010 to 2020. The finding from this study reported an overall level of tooth brushing practice of 12.2%. Thus, this was consistent with the findings of 14.5% in Nigeria^50^ and 15.9% in Sudan^51^.

The current review reported a much lower level of tooth brushing practice than studies across different parts of the world. For instance, a higher level of tooth brushing practice was reported at 77.5% in Kenya^52^, 76.6% in Malawi^53^, 72.4% in Tanzania^54^, 56.5% in Uganda^55^ and 57% in Iran^56^. This variation might be due to variations in scope of the studies and sample size.

Understanding all the variations in the scope and level of emphasis to the assessment of oral hygiene, there exists differences in the level of tooth brushing practice. As an evidence for this is; reports from China and Italy reported a tooth brushing practice of 44.4%^57^ and 33.6%^58^ respectively. Where such variation might be due to differences in population characteristics across the studies.

There exists a variation in the distribution of level of oral hygiene (tooth brushing) across the global population. This uneven distribution to happen to the people might take on difference factors that includes service access differences, awareness level, perception, and healthcare infrastructure. A report from India revealed a level of tooth brushing practice of 99.5%^59^, which is by far higher than the current review report.

With a relative understanding, studies from Eritrea and Sudan reported a tooth brushing practice of 19.1%^60^ and 20%^28^ respectively which is almost comparable with the current review report.

Moreover, an assessment from Saudi Arabia reported the tooth brushing practice of 41.5%^61^. A study from Jorpati, Kathmandu, Nepal also reported the tooth brushing practice of 36.9%^62^. Comparing these findings with the current review report, it is clear that a higher level of oral hygiene practice. A finding from Nigeria also reported a higher level tooth brushing practice which was 28.46%^63^. This variation might be due to differences in scope across the studies.

Furthermore, studies from Malaysia and Tanzania revealed a much higher practice than the current finding. It was 59.4% in Tanzania^64^ and 75.3% in Malaysia^65^. Scope across the studies, sample sizes and population characteristics might have attributed for the observed variations.

### Limitation of the systematic review and meta-analysis

In the current study, tooth brushing was measured two or more times of tooth brushing per day. However, the authors didn’t get a comprehensive expression within the reviewed articles. The authors acknowledge shoratge of related lietertaure while discussing the finding.

## Conclusion

The review reported a lower level of tooth brushing practice in Ethiopia. Strengthening tooth brushing practice in Ethiopia is a key to the promotion of oral hygiene and therefore; concerned bodies Should work on the identified oral health concern of the Ethiopian population.

## Supplementary Information

Supplementary Information.

## Data Availability

The data that supported the finding of this study are available within the manuscript and supplementary file.

## Abbreviations

CI: Confidence interval
MeSH: Medical subject heading
PRISMA: Preferred reporting items for systematic reviews and meta-analyses
