# Supplementary material for: Tooth brushing practice in Ethiopia: a systematic review and meta-analysis
Source: Sci Rep. 2023 Apr 19;13:6418. doi: 10.1038/s41598-023-33541-0 (PMC10115787; doi:10.1038/s41598-023-33541-0)
Supplement: Supplementary file 1 — Supplementary Information. [file 41598_2023_33541_MOESM1_ESM.docx]

| **Section/topic** | **#** | **Checklist item** | **Reported on page #** |
| --- | --- | --- | --- |
| **TITLE** | | |  |
| Title | 1 | Tooth Brushing Practice in Ethiopia: A systematic Review and Meta-Analysis | 1 |
| **ABSTRACT** | | |  |
| Structured summary | 2 | Background: Dental caries is one of the major public health problems affecting 35.3% of the world population by the year 2010. As a preventive mechanism for caries, tooth brushing is one of the common practices to keep oral hygiene. Objective: the current review assessed the state of evidence about tooth brushing practice in Ethiopia from 2010 to 2020. Methods: A systematic literature search was conducted using PubMed, Google Scholar, EMBASE, Hinari and African Journals Online databases. This review restricted the search to studies published in English from 2010 to 2020. The primary outcome for the review was tooth brushing as measured by two or more times of tooth brushing per day. Results: Of the 36 articles identified, ten of which were included in the review. The tooth brushing practice in Ethiopia was low, with variations in the study settings, sample size. Conclusions: Generally, A low tooth brushing practice was reported within the current review. Further research was recommended from this review observation. | 2 |
| **INTRODUCTION** | | |  |
| Rationale | 3 | In Ethiopia the prevalence of dental caries ranges from 21% to 58% whereby pieces of evidence suggested that tooth brushing reduces the likelihood of caries. | 3 |
| Objectives | 4 | To assess the state of evidence about tooth brushing practice in Ethiopia from 2010 to 2020. | 4 |
| **METHODS** | | |  |
| Protocol and registration | 5 | Indicate if a review protocol exists, if and where it can be accessed (e.g., Web address), and, if available, provide registration information including registration number. | Not registered |
| Eligibility criteria | 6 | Study inclusion criteria were the following: (1) study population comprised of any age group, (2) study outcome is tooth brushing, (3) study design is cross-sectional. Exclusion criteria were as follows: (1) qualitative study, (2) report language is non-English, (3) year of publication is older than 2010. | 4 |
| Information sources | 7 | PubMed, Google Scholar, Hinari, EMBASE, and African Journals Online | 4 |
| Search | 8 | Tooth Brushing OR Dental Problems OR Dental Caries) AND Ethiopia. The controlled searches included the following Medical Subject Headings (MeSH) terms: “tooth brushing”, and “Ethiopia” as recommended for each database | 4 |
| Study selection | 9 | literature screening of the extracted articles involved examining the titles and abstracts for relevance | 5 |
| Data collection process | 10 | The reviewer extracted the included data. Information concerning the study characteristics, methodological details, main outcomes, and findings were extracted from the selected articles and organized in an excel table to facilitate the assessment of their quality using Preferred Reporting Items for Systematic Reviews and Meta-Analysis(PRISMA) guidelines. | 5 |
| Data items | 11 | List and define all variables for which data were sought (e.g., PICOS, funding sources) and any assumptions and simplifications made. | NA |
| Risk of bias in individual studies | 12 | For this review, the Joanna Briggs Institutes critical appraisal tool for prevalence study scale was used to assess the risk of bias in cross-sectional studies. A star system was used to assign a rating, where each parameter was awarded a star or two stars for comparability questions and reported as low, medium and good. | 6 |
| Summary measures | 13 | State the principal summary measures (e.g., risk ratio, difference in means). | NA |
| Synthesis of results | 14 | Describe the methods of handling data and combining results of studies, if done, including measures of consistency (e.g., I^2^) for each meta-analysis.  Heterogeneity assessed by suing i^2^ | NA |

Appendix 1: **PRISMA 2009 Checklist**

Page 1 of 2

| **Section/topic** | **#** | **Checklist item** | **Reported on page #** |
| --- | --- | --- | --- |
| Risk of bias across studies | 15 | Specify any assessment of risk of bias that may affect the cumulative evidence (e.g., publication bias, selective reporting within studies).  Risks of bias was also assessed | NA |
| Additional analyses | 16 | Describe methods of additional analyses (e.g., sensitivity or subgroup analyses, meta-regression), if done, indicating which were pre-specified. | NA |
| **RESULTS** | | |  |
| Study selection | 17 | A total of 36 articles were identified from all searches of these 10 met the inclusion criteria and included in the final review | 6 |
| Study characteristics | 18 | Presented in table 1. | 8-9 |
| Risk of bias within studies | 19 | Figure 3 | 8-9 |
| Results of individual studies | 20 | For all outcomes considered (benefits or harms), present, for each study: (a) simple summary data for each intervention group (b) effect estimates and confidence intervals, ideally with a forest plot. | NA |
| Synthesis of results | 21 | Present results of each meta-analysis done, including confidence intervals and measures of consistency. Presented in figure 2 | NA |
| Risk of bias across studies | 22 | Present results of any assessment of risk of bias across studies (see Item 15). | NA |
| Additional analysis | 23 | Give results of additional analyses, if done (e.g., sensitivity or subgroup analyses, meta-regression [see Item 16]).  Subgroup analysis conducted and presented in Table 2 | NA |
| **DISCUSSION** | | |  |
| Summary of evidence | 24 | Presented in discussion part | 10-11 |
| Limitations | 25 | Discuss limitations at study and outcome level (e.g., risk of bias), and at review-level (e.g.,  presented  presented following discussion | 11 |
| Conclusions | 26 | Provide a general interpretation of the results in the context of other evidence, and implications for future research.  Presented in its actual placement | 11 |
| **FUNDING** | | |  |
| Funding | 27 | Describe sources of funding for the systematic review and other support (e.g., supply of data); role of funders for the systematic review.  The authors did not receive any funding source. | NA |

*From:*  Moher D, Liberati A, Tetzlaff J, Altman DG, The PRISMA Group (2009). Preferred Reporting Items for Systematic Reviews and Meta-Analyses:
